# White blood cell count predicts the odds of kidney function decline in a Chinese community-based population

**DOI:** 10.1186/s12882-017-0608-4

**Published:** 2017-06-07

**Authors:** Fangfang Fan, Jia Jia, Jianping Li, Yong Huo, Yan Zhang

**Affiliations:** Department of Cardiology, Peking University First Hospital, No. 8 Xishiku Street, Xicheng District, Beijing, 100034 China

**Keywords:** Chronic inflammation, Chronic renal failure, GFR, Prediction, White blood cell

## Abstract

**Background:**

Inflammatory processes are very important in the development of kidney disease. Nevertheless, the association between white blood cell (WBC) count and the risk of renal dysfunction has not been well-established, especially in subjects without chronic kidney disease (CKD). Our study investigated the association between WBC count and kidney function decline in a Chinese community-based population with baseline estimated glomerular filtration rate (eGFR) ≥60 mL/min/1.73 m^2^.

**Methods:**

A total of 3768 subjects who were enrolled in an atherosclerosis cohort in Beijing were included in this study. EGFRs were calculated at baseline and follow-up using the CKD-EPI formula. The outcomes of this study were renal function decline (RFD) (a drop in eGFR stage along with a decline in eGFR of 25% or exceeding 5 mL/min/1.73 m^2^/year), rapid eGFR decline (an annual decrease in eGFR exceeding 3 mL/min/1.73 m^2^), and incident CKD (eGFR <60 min/1.73 m^2^ at follow-up). Multivariate logistic regression models were used to evaluate the associations between WBC count and each outcome.

**Results:**

On average, the subjects were 56.6 ± 8.5 years old, and 35.9% were male. Of the participants, 48.6% had hypertension and 17.4% had diabetes. The mean (SD) WBC count at baseline was 6.1 ± 1.5 × 10^9^/L. The mean (SD) eGFR at baseline was 101.1 ± 10.6 mL/min/1.73 m^2^. After 2.3 years follow-up, the incidence rates of RFD, rapid eGFR decline and new CKD were 7.7, 20.9, and 0.8%, respectively. WBC count was significantly related to RFD, rapid eGFR decline and new CKD in the univariate analyses. Even after adjustment for demographic variables, comorbidities, medications and baseline eGFR, these associations remained. Moreover, similar trends in RFD were observed in nearly all subgroups stratified by each confounding variable. The increase in the odds of RFD associated with each 10^9^/L increase in WBC count was significantly greater in subjects not undergoing treatment with lipid-lowering drugs than those not undergoing this treatment (P-interaction: 0.05).

**Conclusions:**

In conclusion, elevated WBC count served as a predictor of the odds of kidney function decline in this population, which supports the hypothesis that systemic inflammation may serve as a risk factor for CKD development.

**Electronic supplementary material:**

The online version of this article (doi:10.1186/s12882-017-0608-4) contains supplementary material, which is available to authorized users.

## Background

Chronic kidney disease (CKD) has increasingly become as a worldwide public health problem in the recent decades [1]. As a subgroup with high risk of developing cardiovascular disease (CVD), CKD have been found to share many similar traditional atherosclerosis risk factors with CVD [2, 3]. Currently, CVD is regarded as an inflammatory disease [4, 5], and inflammatory markers are also involved in the development of hypertension [6] and diabetes mellitus [7], potentially indicating that atherosclerosis is a chronic inflammatory process [8]. Therefore, inflammation may also be implicated in the complex pathophysiological mechanisms of CKD, and identifying new modifiable risk factors for CKD at an early stage of kidney disease may help greatly in reducing the burden associated with this illness.

Inflammatory processes have been suspected to play a key role in the development of kidney disease in both animal and human models [9]. Increasing evidence has suggested that several inflammatory markers, including white blood cell (WBC) counts and C-reactive protein (CRP), serum albumin, tumour necrosis factor receptor, ferritin and interleukin-6 levels, may be associated with kidney function and could predict the risk of future kidney function loss and incident CKD [10–18].

However, inconsistent evidence has been reported regarding the relationship between WBC count, which is a traditional indicator of systemic inflammation, and kidney function decline, and this association may be influenced by race and gender [10–15]. Nevertheless, few studies have focused on this topic in Chinese populations [15]. Given the urgency of the epidemiological situation of CKD in China, which may be observed based on CKD prevalence rates that reached as high as 10.8% (119.5 million patients) between 2007 and 2010, as indicated by the results of the National Survey of Chronic Kidney Disease [19], we hoped to determine whether WBC count data were predictive of the odds of future renal function decline in the Chinese population.

Thus, this study aimed to assess the longitudinal associations between WBC count and the decline of kidney function and new-onset of CKD in a Chinese community-based cohort with baseline preserved kidney function.

## Methods

### Study population

The participants included in this study were identified from subjects enrolled in an cohort survey focusing on atherosclerosis diseases conducted from December 2011 to July 2014 in Beijing, China. Detailed study procedures have been described previously [20]. Briefly, 5962 participants aged ≥40 years who had received a baseline survey were invited to participated in a follow-up visit in 2014, 3823 (64.1%) of whom responded on site. The baseline characteristics of non-responders did not differ substantially from those of responders (data not shown). We further excluded participants for whom WBC count measurement data were not obtained at the follow-up visit and those who already had CKD at baseline. Finally, 3768 eligible participants were included in this analysis. This study was approved by the ethics committee of Peking University as well as the ethics committee of Peking University First Hospital, and each participant signed informed consent. We adhered to the principles of the Declaration of Helsinki. The procedures followed were in accordance with institutional guidelines.

### Data collection

As described previously [20], trained research staff collected baseline data according to the standard operating procedure of the study. All participants were interviewed using a standardized questionnaire that included questions about demographic characteristics, health behaviour and medical history. Specifically, current smoker was defined as smoking one cigarette per day for at least half a year, and current drinker was defined as drinking once per week for at least half a year.

Body mass index (BMI) was calculated as weight (kilograms) divided by squared height (meters). Seated brachial blood pressure (BP) of each subject was obtained after a 5-min rest. Calibrated Omron HEM-7117 electronic sphygmomanometers were used to measure BP values. Triplicate measurements were measured on the right arm with at least a 1-min period between successive readings, and their averages were used further.

Blood samples were drawn from each subject’s forearm in the morning after an overnight fasting period. Each patient’s WBC count (×10^9^/L) was measured using a BC-3000 auto haematology analyser (Mindray Medical International, Inc.) in the laboratory of Peking University Shougang Hospital. Baseline fasting blood glucose (FBG), 2 h glucose in the standard 75-g oral glucose tolerance test (OGTT), total cholesterol (TC), low-density lipoprotein (LDL) cholesterol, high-density lipoprotein (HDL) cholesterol, and triglycerides (TG) levels were measured using the Roche C8000 Automatic Analyser.

As described previously [20], serum creatinine levels (μmol/L) at baseline and follow-up were measured using the enzymatic method and Jaffe’s kinetic method, respectively, then transformed using the enzymatic equation, and finally calibrated to the values of one core laboratory. EGFR was estimated using the equation developed by the Chronic Kidney Disease Epidemiology Collaboration (CKD-EPI) [21].

### Outcomes

The Renal function decline (RFD) was the primary outcome in this study. It was defined according to the Kidney Disease: Improving Global Outcome (KDIGO) 2012 definition [22] as follows: “a drop in the GFR category and accompanied by a 25% or greater drop in eGFR from baseline or a sustained decline in eGFR of more than 5 mL/min/1.73 m^2^/year.”

The secondary outcomes in this study were (1) rapid eGFR decline, which was defined as a decline in eGFR exceeding 3 mL/min/1.73 m^2^/year [11, 23], and (2) new-onset CKD, which was defined as an eGFR below 60 mL/min/1.73 m^2^ measured at follow-up survey.

### Statistical analysis

Hypertension was defined as either self-reported hypertension history or a SBP ≥140 mmHg or DBP ≥90 mmHg at baseline. Diabetes mellitus was defined as either self-reported diabetes history or simultaneous identification of an FBG level ≥ 7.0 mmol/L and OGTT value ≥11.1 mmol/L. Dyslipidaemia was defined as any self-reported history of hyperlipidaemia or a TG level ≥ 1.70 mmol/L (150 mg/dL), TC level ≥ 5.18 mmol/L (200 mg/dL), LDL-C level > 3.37 mmol/L (130 mg/dL) or HDL-C level < 1.04 mmol/L (40 mg/dL). CVD was defined as any self-reported suffering from coronary heart disease, stroke, or transient ischaemic attack previously.

Data were expressed as the mean (standard deviation, SD) for continuous variables and percentages (%) for categorical variables. Normally distributed continuous variables were compared using one-way ANOVA tests. Categorical variables were compared using the Pearson χ2 test. WBC count was examined as both continuous and categorical (quartiles) variables. Univariate and multivariate Logistic regression models were generated to investigate the effects of WBC count on the occurrence of all outcomes. Covariates were selected based on previous studies relating to WBC count or renal function. The odds of renal outcomes were evaluated in association with 10^9^/L increases and quartiles of WBC count.

A subgroup analysis was performed to examine the associations between WBC count and the odds of all outcomes following adjustment for sex, age, BMI, baseline eGFR, current smoking, current drinking, hypertension, diabetes mellitus, dyslipidaemia, history of CVD, antihypertensive drug use, hypoglycaemic drug use, and lipid-lowering drug use. Interaction terms were entered into the logistic regression models to compare the effects of WBC count on each outcome between the analysed subgroups.

We also performed sensitivity analyses excluding subjects with WBC counts not in the normal range (4.0–10.0 × 10^9^/L), reflecting the possible influence of acute infectious diseases and haematological system diseases.

A *P*-value of <0.05 (two-sided) was considered statistically significant for all tests. All analyses were performed using Empower(R) (www.empowerstats.com, X&Y solutions, Inc. Boston MA) and R (http://www.R-project.org).

## Results

Table 1 showed the baseline characteristics of the participants overall and stratified by WBC count quartiles. The mean age of the participants was 56.6 ± 8.5 years. Of the participants, 35.9% were male, and the mean (SD) BMI was 26.0 ± 3.4 kg/m^2^. Hypertension was present in 48.6% of all participants, whereas 17.4 and 75.1% of the participants had diabetes mellitus and dyslipidaemia, respectively. The prevalence of CVD was 12.3%. The mean (SD) baseline WBC count was 6.1 ± 1.5 × 10^9^/L, and the mean (SD) baseline eGFR was 101.1 ± 10.6 mL/min/1.73 m^2^. Subjects with higher WBC counts were more likely to be male; had significantly higher BMIs; had higher prevalence rates of hypertension, diabetes mellitus, dyslipidaemia, and CVD; more frequently used antihypertensive drugs, hypoglycaemic drugs and lipid-lowering drugs; and had lower baseline eGFR levels.

**Table 1:** Baseline characteristics of the study cohort

Variables	White blood cell, 10^9^/L					
	All	Quartile 1 (<5.0)	Quartile 2 (5.0–6.0)	Quartile 3 (6.0–7.0)	Quartile 4 (≥7.0)	P-trend
	*N* = 3768	*N* = 835	*N* = 1047	*N* = 925	*N* = 961	
Age, year	56.6 (8.5)	56.3 (8.0)	56.5 (8.5)	56.9 (8.5)	56.8 (9.0)	0.149
Males, *n* (%)	1352 (35.9%)	203 (24.3%)	356 (34.0%)	336 (36.3%)	457 (47.6%)	<0.001
White blood cell, 10^9^/L	6.1 (1.5)	4.4 (0.5)	5.5 (0.3)	6.4 (0.3)	8.2 (1.2)	<0.001
Body mass index, kg/m^2^	26.0 (3.4)	25.3 (3.3)	25.9 (3.2)	26.2 (3.4)	26.6 (3.4)	<0.001
eGFR, mL/min/1.73m^2^	101.1 (10.6)	101.9 (10.1)	101.4 (10.0)	101.0 (10.6)	100.2 (11.8)	0.001
Current smoking status, *n* (%)	709 (18.8%)	82 (9.8%)	164 (15.7%)	168 (18.2%)	295 (30.7%)	<0.001
Current drinking status, *n* (%)	868 (23.0%)	145 (17.4%)	257 (24.5%)	221 (23.9%)	245 (25.5%)	<0.001
Prevalence of disease, *n* (%)						
Hypertension	1833 (48.6%)	355 (42.5%)	500 (47.8%)	466 (50.4%)	512 (53.3%)	<0.001
Diabetes mellitus	654 (17.4%)	105 (12.6%)	153 (14.6%)	167 (18.1%)	229 (23.8%)	<0.001
Dyslipidemia	2768 (75.1%)	567 (69.6%)	739 (72.3%)	709 (78.4%)	753 (79.8%)	<0.001
Cardiovascular disease	477 (12.7%)	82 (9.8%)	119 (11.4%)	132 (14.3%)	144 (15.0%)	<0.001
Treatment, n (%)						
Antihypertensive	1183 (31.6%)	197 (23.8%)	323 (31.0%)	315 (34.2%)	348 (36.4%)	<0.001
Hypoglycemic	382 (10.2%)	62 (7.4%)	88 (8.4%)	105 (11.4%)	127 (13.3%)	<0.001
Lipid-lowering	397 (10.6%)	59 (7.1%)	104 (10.0%)	109 (11.9%)	125 (13.1%)	<0.001

*Abbreviations*: *eGFR* estimated glomerular filtration rate

After a mean follow-up of 2.4 years (median: 2.3; 25th centile-75th centile: 2.1–2.6), the incidence rates of RFD, rapid eGFR decline and new CKD were 7.7, 20.9, and 0.8%, respectively. The odds of RFD, rapid eGFR decline and new CKD gradually increased with increasing WBC counts (Table 2).

**Table 2:** Renal function decline, rapid eGFR decline and incident CKD according to white blood cell quartiles

White blood cell	Renal function decline	Rapid eGFR decline	Incident CKD
All	292 (7.7%)	787 (20.9%)	30 (0.8%)
Quartile 1	52 (6.2%)	153 (18.3%)	3 (0.4%)
Quartile 2	67 (6.4%)	205 (19.6%)	5 (0.5%)
Quartile 3	73 (7.9%)	195 (21.1%)	6 (0.6%)
Quartile 4	100 (10.4%)	234 (24.3%)	16 (1.7%)
P-trend	<0.001	0.001	0.002

*Abbreviations*: *eGFR* estimated glomerular filtration rate, *CKD* chronic kidney disease

TThe effects of WBC count on all outcomes in the multivariate regression analysis are displayed in Table 3. The odds of RFD, rapid eGFR decline and new CKD were positively related to WBC count, and these relationships remained statistically significant after adjustment for age, sex, baseline eGFR and various other baseline parameters. ORs (95% confidence interval, CI) of 1.14 (1.06, 1.23), 1.11 (1.05, 1.17) and 1.36 (1.11, 1.68) were identified in association with each 10^9^/L increase in WBC count for RFD, rapid eGFR decline and new CKD, respectively. The odds of RFD, rapid eGFR decline and new CKD also demonstrated consistent dose-dependent relationships with the WBC count quartiles (OR [95% CI] for highest versus lowest quartile: 2.01 [1.38, 2.93]; 1.49 [1.17, 1.91]; 4.79 [1.00, 23.00]; P-trend: <0.001; <0.001; 0.018).

**Table 3:** Multivariate regression for the effect of white blood cell on renal function decline, rapid eGFR decline and incident CKD

Variables	Crude model	Adjusted model 1	Adjusted model 2
	OR (95% CI)	OR (95% CI)	OR (95% CI)
Renal function decline			
WBC continuous, 10^9^/L	1.12 (1.04, 1.20)	1.14 (1.06, 1.22)	1.14 (1.06, 1.23)
WBC quartiles			
Quartile 1	1(Reference)	1(Reference)	1(Reference)
Quartile 2	1.03 (0.71, 1.50)	1.06 (0.73, 1.54)	1.07 (0.72, 1.60)
Quartile 3	1.29 (0.89, 1.87)	1.34 (0.92, 1.94)	1.45 (0.99, 2.15)
Quartile 4	1.75 (1.23, 2.48)	1.89 (1.32, 2.69)	2.01 (1.38, 2.93)
P-trend	<0.001	<0.001	<0.001
Rapid eGFR decline			
WBC continuous, 10^9^/L	1.10 (1.04, 1.15)	1.12 (1.06, 1.18)	1.11 (1.05, 1.17)
WBC quartiles			
Quartile 1	1(Reference)	1(Reference)	1(Reference)
Quartile 2	1.09 (0.86, 1.37)	1.12 (0.89, 1.42)	1.11 (0.87, 1.41)
Quartile 3	1.19 (0.94, 1.51)	1.24 (0.98, 1.57)	1.20 (0.94, 1.54)
Quartile 4	1.43 (1.14, 1.80)	1.58 (1.25, 1.99)	1.49 (1.17, 1.91)
P-trend	0.001	<0.001	<0.001
Incident CKD			
WBC continuous, 10^9^/L	1.41 (1.20, 1.64)	1.33 (1.09, 1.62)	1.36 (1.11, 1.68)
WBC quartiles			
Quartile 1	1(Reference)	1(Reference)	1(Reference)
Quartile 2	1.33 (0.32, 5.58)	1.35 (0.30, 6.02)	1.72 (0.29, 10.25)
Quartile 3	1.81 (0.45, 7.26)	1.73 (0.41, 7.37)	2.11 (0.38, 11.68)
Quartile 4	4.70 (1.36, 16.17)	3.30 (0.90, 12.09)	4.79 (1.00, 23.00)
P-trend	0.002	0.031	0.018

**Model 1:** adjusted for age, sex, and eGFR at baseline

**Model 2:** adjusted for variables in model 1 and body mass index, current smoking, current drinking, hypertension, diabetes mellitus, dyslipidemia, history of cardiovascular disease, antihypertensive drugs, hypoglycemic drugs, and lipid-lowering drugs

*Abbreviations*: *WBC* white blood cell, *eGFR* estimated glomerular filtration rate, *CKD* chronic kidney disease, *OR* odd ratio, *CI* confidence interval

The results of the subgroup analyses are presented in Table 4. No significant heterogeneity was observed across nearly all of the subgroups defined according to sex; age (<60 or ≥60-year-old); BMI (<28 or ≥28 kg/m^2^); baseline eGFR (<the median value of 102.5 or ≥102.5 mL/min/1.73 m^2^); current smoking; current drinking; prevalence of hypertension, diabetes mellitus, dyslipidaemia and CVD; and usage of antihypertensive drugs, hypoglycaemic drugs and lipid-lowering drugs. However, the increase in the odds of rapid eGFR decline associated with each 10^9^/L increase in WBC was significantly greater in older than younger subjects (P-interaction = 0.04). Meanwhile, the increases in the odds of RFD (OR = 1.18, 95% CI: 1.09–1.28, P-interaction = 0.05), rapid eGFR decline (OR = 1.13, 95% CI: 1.07–1.20, P-interaction = 0.09) and new CKD (OR = 1.54, 95% CI: 1.20–1.96, P-interaction = 0.07) associated with each 10^9^/L increase of WBC count were significantly greater in those not undergoing treatment with lipid-lowering drugs than those undergoing such treatment; however, the *P*-values for these interactions were only borderline significant.

**Table 4:** Subgroup analysis for effect of white blood cell on risks of renal function decline, rapid eGFR decline or incident CKD

Variable	Renal Function Decline			Rapid eGFR Decline			Incident CKD		
	Incidence, *n*(%)	OR (95% CI)	P-interaction	Incidence, *n*(%)	OR (95% CI)	P-interaction	Incidence, *n*(%)	OR (95% CI)	P-interaction
Sex									
Male	88 (6.5%)	1.10 (0.97, 1.26)	0.5244	244 (18.0%)	1.11 (1.01, 1.21)	0.9709	9 (0.7%)	1.57 (1.06, 2.32)	0.4362
Female	204 (8.4%)	1.16 (1.06, 1.27)		543 (22.5%)	1.11 (1.04, 1.18)		21 (0.9%)	1.30 (1.02, 1.66)	
Age, years									
< 60	195 (7.4%)	1.10 (1.00, 1.21)	0.1517	543 (20.5%)	1.07 (1.00, 1.14)	0.0398	7 (0.3%)	1.51 (1.00, 2.27)	0.5985
≥ 60	97 (8.6%)	1.23 (1.09, 1.39)		244 (21.7%)	1.20 (1.09, 1.32)		23 (2.0%)	1.32 (1.03, 1.69)	
BMI, kg/m^2^									
< 28	212 (7.6%)	1.13 (1.03, 1.23)	0.6299	577 (20.6%)	1.12 (1.06, 1.19)	0.4084	23 (0.8%)	1.30 (1.02, 1.67)	0.4080
≥ 28	80 (8.3%)	1.18 (1.02, 1.36)		210 (21.7%)	1.07 (0.96, 1.18)		7 (0.7%)	1.61 (1.04, 2.47)	
Baseline eGFR, mL/min/1.73 m^2^									
Low (<102.5)	166 (8.8%)	1.18 (1.07, 1.31)	0.2632	356 (18.9%)	1.13 (1.05, 1.22)	0.4544	28 (1.5%)	1.38 (1.11, 1.72)	0.8913
High (≥102.5)	126 (6.7%)	1.09 (0.97, 1.22)		430 (22.8%)	1.09 (1.01, 1.17)		2 (0.1%)	1.31 (0.57, 2.97)	
Current smoking									
No	247 (8.1%)	1.16 (1.07, 1.27)	0.3243	677 (22.1%)	1.11 (1.05, 1.18)	0.9003	27 (0.9%)	1.37 (1.10, 1.70)	0.9942
Yes	45 (6.3%)	1.06 (0.89, 1.26)		110 (15.5%)	1.10 (0.98, 1.24)		3 (0.4%)	1.37 (0.76, 2.48)	
Current drinking									
No	238 (8.2%)	1.17 (1.08, 1.28)	0.1173	640 (22.0%)	1.12 (1.05, 1.19)	0.4544	25 (0.9%)	1.36 (1.08, 1.72)	0.9549
Yes	54 (6.2%)	1.01 (0.84, 1.21)		147 (16.9%)	1.06 (0.95, 1.20)		5 (0.6%)	1.38 (0.86, 2.22)	
Hypertension									
No	112 (5.8%)	1.12 (0.99, 1.26)	0.6203	346 (17.9%)	1.07 (0.99, 1.16)	0.2295	4 (0.2%)	1.16 (0.67, 2.02)	0.4416
Yes	180 (9.8%)	1.16 (1.05, 1.28)		441 (24.1%)	1.14 (1.06, 1.22)		26 (1.4%)	1.44 (1.12, 1.84)	
Diabetes mellitus									
No	207 (6.6%)	1.12 (1.02, 1.23)	0.5148	570 (18.3%)	1.11 (1.05, 1.18)	0.7693	18 (0.6%)	1.45 (1.08, 1.94)	0.5844
Yes	85 (13.0%)	1.18 (1.04, 1.34)		217 (33.2%)	1.09 (0.99, 1.21)		12 (1.8%)	1.29 (0.94, 1.76)	
Dyslipidemia									
No	68 (7.4%)	1.19 (1.02, 1.39)	0.5331	186 (20.3%)	1.10 (0.99, 1.22)	0.8334	5 (0.5%)	1.62 (0.95, 2.75)	0.5003
Yes	215 (7.8%)	1.13 (1.03, 1.23)		583 (21.1%)	1.11 (1.05, 1.18)		25 (0.9%)	1.32 (1.05, 1.67)	
Cardiovascular disease									
No	240 (7.3%)	1.14 (1.05, 1.24)	0.9617	657 (20.0%)	1.10 (1.04, 1.17)	0.6392	18 (0.5%)	1.44 (1.09, 1.89)	0.5951
Yes	52 (10.9%)	1.14 (0.96, 1.35)		130 (27.3%)	1.14 (1.00, 1.30)		12 (2.5%)	1.28 (0.91, 1.79)	
Antihypertensive drugs									
No	165 (6.4%)	1.12 (1.02, 1.24)	0.5696	477 (18.6%)	1.08 (1.01, 1.16)	0.2454	7 (0.3%)	1.06 (0.66, 1.70)	0.2017
Yes	125 (10.6%)	1.17 (1.04, 1.31)		305 (25.8%)	1.15 (1.06, 1.26)		22 (1.9%)	1.47 (1.16, 1.86)	
Hypoglycemic drugs									
No	239 (7.1%)	1.14 (1.04, 1.24)	0.7537	648 (19.2%)	1.11 (1.05, 1.18)	0.8791	20 (0.6%)	1.52 (1.16, 2.00)	0.2124
Yes	52 (13.6%)	1.17 (0.99, 1.38)		138 (36.1%)	1.10 (0.97, 1.25)		10 (2.6%)	1.12 (0.72, 1.74)	
Lipid-lowering drugs									
No	255 (7.6%)	1.18 (1.09, 1.28)	0.0541	679 (20.4%)	1.13 (1.07, 1.20)	0.0872	24 (0.7%)	1.54 (1.20, 1.96)	0.0677
Yes	34 (8.6%)	0.94 (0.74, 1.19)		102 (25.7%)	0.99 (0.86, 1.15)		4 (1.0%)	0.84 (0.40, 1.75)	

**Variables in the model:** age, sex, eGFR at baseline, body mass index, current smoking, current drinking, hypertension, diabetes mellitus, dyslipidemia, history of cardiovascular disease, antihypertensive drugs, hypoglycemic drugs, and lipid-lowering drugs

*Abbreviations*: *eGFR* estimated glomerular filtration rate, *CKD* chronic kidney disease, *OR* odd ratio, *CI* confidence interval

The results of the sensitivity analysis in which subjects with WBC count not in the normal range (4.0–10.0 × 10^9^/L) were excluded are shown in Additional file 1: Table S1. Similar results were identified in this sensitivity analysis.

## Discussion

The main findings of our study were as follows: WBC count, a traditional indicator of systemic inflammation, was significantly associated with the odds of future RFD, rapid eGFR decline and incident CKD even after adjusting for known atherosclerosis risk factors such as age, sex, BMI, lifestyles, comorbidities, and medications, and baseline eGFR in this Chinese community-based population with preserved kidney function (eGFR ≥60 mL/min/1.73 m^2^) at baseline. After a mean follow-up 2.4 years, subjects with the highest quartile of WBC count were at 1.49–fold greater odds of rapid eGFR decline, 2.01–fold greater odds of RFD, and 4.79-fold greater odds of new incident CKD. Detection of higher WBC counts in an early stage may be valuable for CKD risk assessment and prevention.

Low-grade inflammation has been found to be common in patients with CKD and end-stage renal disease (ESRD) and has already been recognized as playing a unique role in the pathophysiology and taking account for the future CVD and all-cause mortality risk [24]. However, inconsistent evidence has been presented regarding whether inflammation markers, including WBC count, may be used to predict the risk of future kidney function decline. In the NHANES II, a nationally representative cohort of 9250 US adults with 17 years’ follow-up, WBC counts were found to be positive related to the risk of future CKD, defined as ESRD treatment or death due to kidney disease (relative hazard, 2.01, 95% CI, 1.11 to 3.65 for the highest versus lowest quartile) [10]. Additionally, data from the Cardiovascular Health Study (CHS) revealed that higher WBC counts could independently predict annual increases in serum creatinine or declines in eGFR in a community-based cohort of 5888 elderly individuals with 4 to 7 years’ follow-up [11]. Similar findings were also identified in data from the following two long-term population-based cohorts in the US: 14,854 middle-aged adults from 4 different communities with a mean follow-up of almost 14 1/2 years (ARIC) [12] and 4926 subjects from the Beaver Dam CKD study with 15 years of follow-up [13].

However, in the ARIC study, WBC count was found to be a better predictor of CKD risk stronger in African Americans relative to whites (Hazard Ratios [95% CI]: 1.14 [1.07–1.23] vs. 1.10 [1.04–1.17] for 1-SD increment, P-interaction = 0.08 for continuous WBC count and 0.004 for race-specific WBC quartiles) [12]. Furthermore, sex differences for the association between WBC count and CKD were identified in a cohort in 2825 Korean adults from the 2007 Korean National Health and Nutrition Examination Survey. In this study, a significant association was only identified in women [14]. In addition, high sensitivity CRP and ferritin levels but not WBC count were found to be associated with increased odds of both renal replacement therapy and rapid renal decline in 3303 Chinese patients with stage 3–5 CKD after a mean follow-up period of 3.2 years [15]. The reason for the identification of inconclusive results in the aforementioned studies might be the significantly different populations studied.

Until now, the potential mechanisms by which inflammation processes lead to the development of CKD were not clear. Evidence has shown that inflammation has been hypothesized to play an important role in systemic atherosclerosis development [4, 5, 8]. Some may speculate that WBC counts are predictive of the risk of future CKD because of their relationships with many other atherosclerosis risk factors, like smoking, diabetes or hypertension [6, 7]. Adipose tissue can also be regarded as a source of inflammation [25]. Given the amount of literature regarding the associations between elevated WBC count and atherosclerotic disease and atherosclerotic disease and CKD, the positive relationship observed between WBC count and kidney function decline in our study may indicate association but not necessarily causation. However, the main results were not changed or weakened after further adjusting for confounders, including smoking status, BMI, hypertension, diabetes and CVD. Additionally, many animal and in vitro inflammation experiments have provided pathways through which the inflammation process may play important roles in CKD development [26–28]. Nevertheless, there may be other parameters related to inflammation, atherosclerosis and CVD that are potential confounders not adjusted for in this study. Therefore, the predictive value of WBC count should be more comprehensively verified in other large populations, and the precise pathological mechanisms behind this association should be investigated in further studies.

In CKD patients, several interventions, such as general lifestyle modifications, dietary supplementation and pharmacological agents, have already been proposed to target inflammation [23, 29]. However, it is not clear whether these therapies can prevent kidney function decline at a stage of kidney disease at which CKD has not yet occurred. Statins, as important agents with plenty of evidence supporting their role in CVD prevention and treatment, may play an efficient role in terms of not only lipid-lowering but also anti-inflammatory effect. Based on this theory, CARE trial investigators performed a post hoc analysis to examine the association between pravastatin use, inflammatory markers and the rate of kidney function decline, and found that the rate of renal function loss was 0.8 mL/min/1.73 m^2^/year lower in pravastatin than placebo group (*P* = 0.039); this study followed 108 subjects for 58 months, and the results suggested that those with the highest level of inflammatory markers derived greater renal benefits from pravastatin than did those with low levels (P-interaction = 0.047) [30]. Likewise, the results of the LORD trial, an RCT including 117 patients with serum creatinine levels >120 μml/L who were followed for an average of 2.5 years, demonstrated that CKD patients with high levels of inflammatory makers who were treated with atorvastatin had significantly lower eGFR declines [31]. Similarly, our data also suggested that subject using lipid-lowering drugs were more likely to have lower odds of future RFD, rapid eGFR decline and new CKD for each 10^9^/L increase in WBC count than were those not using these drugs, although the P-interaction were borderline significance. However, the results of the SHARP study suggested that lowering LDL-C by approximately 1 mmol/L for approximately 5 years through the use of lipid-lowering treatment had no significant effect on the rate of eGFR change among 6245 non-dialysis participants with CKD; however, the effect of this treatment in patients with elevated inflammatory markers was not evaluated and worthy of further exploration [32].

Our study has several limitations. First, we did not assess other more sensitive markers of inflammation, such as the erythrocyte sedimentation rate or high sensitivity CRP, tumour necrosis factor-alpha and plasminogen activator inhibitor-1 levels at baseline [33], and related analyses were not available. Second, urinary albumin was used to define CKD, which has already been recognized as an important predictor of kidney function decline especially, in diabetes patients. We did not examine the albumin-creatinine ratio or urine dipstick results at baseline and, therefore, were not able to evaluate these factors, and we could not exclude patients with eGFRs ≥60 mL/min/1.73 m^2^ but abnormal urinary albumin levels from our analysis. However, no significant heterogeneity was identified in subgroup analysis according to diabetes status. Third, WBC count can be affected by acute infectious diseases; chronic inflammation processes; haematological system diseases; nutritional status; allergies; and medications, such as glucocorticoids, and these may have served as confounding factors. Therefore, we further performed a sensitivity analysis including a subset of patients with WBCs in the normal range, and the results were unchanged. Fourth, monocyte count has been found to be related to the risk of incident CKD and progression to ESRD [34]. However, we did not have the data on monocyte count and were not able to perform related analyses. Lastly, our study included only Chinese participants and may not be generalizable to other race-ethnic populations.

## Conclusions

In conclusion, the results of our study demonstrate that WBC count, a traditional indicator of systemic inflammation, may predict the odds of future kidney function decline in a Chinese community-based population with normal kidney function. Prospective assessment of WBC count may help to promote the recognition of patients at high risk of kidney function decline. Interventions that may potentially reduce inflammation might also confer potential renal benefits. Further studies should focus on the precise pathological mechanisms behind this association and take inflammation as a treatment target to investigate its potential preventive effects on kidney disease.

## Additional file

Additional file 1: Table S1. Multivariate regression for the effect of white blood cell on renal function decline, rapid eGFR decline and incident CKD in subjects with WBC count in normal range (DOC 48 kb)

## Abbreviations

BMI: Body mass index
BP: Blood pressure
CKD: Chronic kidney disease
CVD: Cardiovascular disease
HDL: High-density lipoprotein
LDL: Low-density lipoprotein
OGTT: 75-g oral glucose tolerance test
RFD: Renal function decline
TC: Total cholesterol
TG: Triglycerides
WBC: White blood cell
