# Supplementary material for: White blood cell count predicts the odds of kidney function decline in a Chinese community-based population
Source: BMC Nephrol. 2017 Jun 7;18:190. doi: 10.1186/s12882-017-0608-4 (PMC5463367; doi:10.1186/s12882-017-0608-4)
Supplement: Additional file 1: Table S1. — Multivariate regression for the effect of white blood cell on renal function decline, rapid eGFR decline and incident CKD in subjects with WBC count in normal range (DOC 48 kb) [file 12882_2017_608_MOESM1_ESM.doc]

# Supplementary Data:

**Table S1. Multivariate regression for the effect of white blood cell on renal function decline, rapid eGFR decline and incident CKD in subjects with WBC count in normal range**

| Variables | Crude model | Adjusted model 1 | Adjusted model 2 |
| --- | --- | --- | --- |
|  | OR (95%CI) | OR (95%CI) | OR (95%CI) |
| **Renal function decline** | | | |
| WBC continuous, 10^9^/L | 1.14 (1.04, 1.25) | 1.16 (1.06, 1.27) | 1.18 (1.07, 1.30) |
| WBC quartiles |  |  |  |
| Quartile 1 (4.0-5.1) | 1(Reference) | 1(Reference) | 1(Reference) |
| Quartile 2 (5.1-6.0) | 1.02 (0.69, 1.50) | 1.06 (0.72, 1.56) | 1.13 (0.75, 1.71) |
| Quartile 3 (6.0-7.0) | 1.28 (0.88, 1.86) | 1.33 (0.91, 1.93) | 1.50 (1.01, 2.24) |
| Quartile 4 (7.0-10.0) | 1.70 (1.18, 2.43) | 1.83 (1.27, 2.64) | 2.04 (1.38, 3.01) |
| P-trend | 0.001 | <0.001 | <0.001 |
| **Rapid eGFR decline** | | | |
| WBC continuous, 10^9^/L | 1.10 (1.04, 1.17) | 1.13 (1.06, 1.20) | 1.11 (1.04, 1.19) |
| WBC quartiles |  |  |  |
| Quartile 1 (4.0-5.1) | 1(Reference) | 1(Reference) | 1(Reference) |
| Quartile 2 (5.1-6.0) | 1.11 (0.87, 1.41) | 1.14 (0.89, 1.45) | 1.15 (0.89, 1.48) |
| Quartile 3 (6.0-7.0) | 1.22 (0.96, 1.55) | 1.26 (0.99, 1.60) | 1.24 (0.96, 1.59) |
| Quartile 4 (7.0-10.0) | 1.41 (1.12, 1.79) | 1.53 (1.20, 1.95) | 1.48 (1.15, 1.91) |
| P-trend | 0.003 | <0.001 | 0.002 |
| **Incident CKD** | | | |
| WBC continuous, 10^9^/L | 1.50 (1.15, 1.95) | 1.32 (1.00, 1.74) | 1.42 (1.04, 1.94) |
| WBC quartiles |  |  |  |
| Quartile 1 (4.0-5.1) | 1(Reference) | 1(Reference) | 1(Reference) |
| Quartile 2 (5.1-6.0) | 1.42 (0.34, 5.95) | 1.59 (0.36, 7.03) | 1.97 (0.33, 11.65) |
| Quartile 3 (6.0-7.0) | 1.73 (0.43, 6.92) | 1.82 (0.43, 7.70) | 2.26 (0.42, 12.28) |
| Quartile 4 (7.0-10.0) | 3.91 (1.11, 13.77) | 3.08 (0.83, 11.48) | 4.40 (0.91, 21.35) |
| P-trend | 0.013 | 0.066 | 0.040 |

**Model 1:** adjusted for age, sex, and eGFR at baseline;

**Model 2:** adjusted for variables in model 1 and body mass index, current smoking, current drinking, hypertension, diabetes mellitus, dyslipidemia, history of cardiovascular disease, antihypertensive drugs, hypoglycemic drugs, and lipid-lowering drugs.

**Abbreviations:** WBC, white blood cell; eGFR, estimated glomerular filtration rate; CKD, chronic kidney disease; OR, odd ratio; CI, confidence interval.
